# Systematic review of information and support interventions for caregivers of people with dementia

**DOI:** 10.1186/1471-2318-7-18

**Published:** 2007-07-27

**Authors:** Carl A Thompson, Karen Spilsbury, Jill Hall, Yvonne Birks, Colin Barnes, Joy Adamson

**Affiliations:** 1 Department of Health Sciences, University of York, York, UK; 2 Speech and Language Therapy Department, St James Hospital, Portsmouth, UK

## Abstract

**Background:**

Dementia is an important health and social care problem and is one of the main causes of disability in later life. The number of families affected by dementia will dramatically increase over the next five decades. Despite the implications for health and social care services in the future, the overwhelming majority of care for people with dementia takes place away from health care settings. Providing informal care for someone with dementia can be psychologically, physically and financially expensive and a range of health service interventions aimed at supporting and providing information to these carers has developed to help carers meet these demands. This review examines whether information and support interventions improve the quality of life of people caring for someone with dementia.

**Methods:**

A systematic review examining evidence from randomised controlled trials in which technology, individualised or group-based interventions built around the provision of support and/or information were evaluated.

**Results:**

Forty-four studies were included in the review. Controlling for the quality of the evidence, we found statistically significant evidence that group-based supportive interventions impact positively on psychological morbidity. However, whilst the improvement was unlikely to be due to chance, the clinical significance of this finding should be interpreted tentatively, due to the difficulties in interpreting the standardised mean difference as a measure of effect and the complex aetiology of depression. No evidence was found for the effectiveness of any other form of intervention on a range of physical and psychological health outcomes.

**Conclusion:**

There is little evidence that interventions aimed at supporting and/or providing information to carers of people with dementia are uniformly effective. There is a pressing need to ensure that supportive interventions at the development stage are accompanied by good quality randomised evaluations in which outcomes that are important to clinicians and carers are measured.

## Background

There are an estimated 7.4 million people living with dementia worldwide and at least 3.7 million with Alzheimer's disease (AD) [1,2]. In the UK dementia affects 1.1% of the population (683,597 people) [3]. The annual total cost of care for people with late onset dementia in the UK for 2005/6 is estimated to be over €17 billion: this equates to €25,472 per person [3]. In the US, estimated annual costs are $8,064 for formal services and $23,436 for informal care [4]. Informal caregiving constitutes a significant proportion of the care provided to people with dementia. Informal caregivers can be defined as those individuals who provide extraordinary, uncompensated care, predominantly in the home. The role, carried out primarily by family members, involves committing significant amounts of time and energy over months or years, and requires the performance of tasks that may be physically, emotionally, socially, and financially demanding [5]. There are at least 6 million informal caregivers in the UK [6] and an estimated 22 million in the US [7]. Caring for a family member with dementia is stressful and burdensome, and caregivers' physical and emotional health often suffers as a result [8]. Carer burden is associated with several factors including the relationship to the care recipient, gender, age, ethnicity and care recipient behavioural problems [9]. It is likely then that no single supportive intervention "fits all" caregivers.

A variety of interventions have been developed which aim to offer support for caregivers. Interventions include: training and education programmes, information-technology based support including specialized computer and telephone networks, and formal approaches to planning care which take into account the specific needs of caregivers and people with dementia sometimes using specially designated nurses or other members of the healthcare team. Existing reviews of interventions have tended to focus either on a particular intervention type, for example psychosocial interventions [10,11], or particular outcomes, such as carer burden [12,13]. This systematic review aims to assess the effectiveness of interventions based around information and support provision for informal caregivers of people with dementia in community settings. It is worth noting that our focus was on health service interventions rather than the professional group responsible for the intervention delivery. Consequently, whilst the majority of interventions are professionally delivered other non health care professionals may also be involved.

## Methods

### Inclusion criteria

Studies were selected for inclusion if they met pre-specified criteria (Table 1).

**Table 1:** Inclusion criteria

Study design:	randomised controlled trials.
Study participants:	principal informal caregiver (not a paid professional) and care recipient (diagnosed with dementia) dyad living in the community.
Intervention:	information and/or support intervention.
Outcomes:	*caregiver outcomes*: quality of life, physical and mental health, burden or satisfaction; *and patient outcomes*: such as activities of daily living or behaviours; *health service utilization*: such as numbers of in-patient, outpatient or primary health care contacts; *economic outcomes*: such as time spent on caring activities.

### Search Strategy

Trials were identified from a search of the Specialized Register of the Cochrane Dementia and Cognitive Improvement Group during November 2003 and October 2005 using the terms: computer*, telephon*, training*, eduation*, information, "care-planning", carer*, caregiv*. Databases included in the Specialized Register at that time are detailed in Table 2. No language restrictions were imposed. Citation searches for key papers, reference checking and contact with authors to obtain further details (where necessary) were all undertaken. Thus, our search strategy was iterative and multi-stage involving computerised, hand and personal contact searching.

**Table 2:** Databases searched

Cochrane Central Register of Controlled Trial
Current Controlled trials
ClinicalTrials.gov
MEDLINE
EMBASE
PsycINFO
CINAHL (Cumulative Index to Nursing and Allied Health Literature)
SIGLE (Grey Literature in Europe)
ISTP (Index to Scientific and Technical Proceedings)
INSIDE (BL database of Conference Proceedings and Journals)
Aslib Index to Theses (UK and Ireland theses)
Dissertation Abstract (USA)
ADEAR (Alzheimer's Disease Clinical Trials Database)
Alzheimer Society
South Australian Network for Research on Ageing
US Dept of Veterans Affairs Cooperative Studies
National Institutes of Health (NIH)
GlaxoSmithKline
Schering Health Care Ltd
Hong Kong Health Services Research Fund
Medical Research Council (MRC)
National Research Register
NHS R&D Health Technology Assessment Programme
LILACS:Latin American and Carribbean Health Science Literature

### Study selection and data extraction

A single author discarded publications deemed irrelevant on the basis of title and/or abstract. From the refined list of citations, at least two authors independently selected the trials for inclusion if they met the necessary quality criteria. Any disagreements were resolved with a third party. Summary statistics for each trial and each outcome were collected. For continuous data these were mean change from baseline, standard error of mean change, and number of patients for each treatment group at each assessment. Where changes from baseline were not reported, the mean, standard deviation and the number of people in each treatment arm at each time point were extracted (if available). For binary data, the numbers in each treatment group and numbers experiencing the outcome of interest were sought. To allow an intention-to-treat (ITT) analysis, the data was sought irrespective of compliance, whether or not the person was subsequently deemed ineligible, or otherwise excluded from treatment or follow-up. If ITT data were not available in the publications, 'on-treatment' or the data of those who completed the trial were sought and indicated as such. In studies where a cross-over design was used, only data from the first treatment phase after randomisation were eligible for inclusion.

### Quality assessment

Each of the authors assessed the studies according to agreed criteria [14,15] and included the adequacy of the randomisation process in each trial (Table 3). Because allocation systems have the potential to increase bias in studies [16] only those studies that were adequate or unclear were included in the review. Other elements of study quality noted, but not scored, were: 'blinding' of participants and outcome raters; level of caregiver drop-out at study follow-up stage; follow-up analysis of people leaving the study and; equal treatment of both intervention and control participants in all respects other than the delivery of the supportive intervention.

**Table 3:** Adequacy of the randomisation process

**1) Adequate**
i) a central allocation process by an office or third party unaware of subject characteristics
ii) pre-numbered packages of support administered to carers sequentially
iii) use of an on-site or coded computer system with a locked, unreadable file with allocation occurring only after carers or patient details are inputted
iv) assignment via scaled, numbered and opaque envelopes
v) other combinations which provide evidence of adequacy in concealment

**2) Unclear**
i) study used list or tables to allocate assignments
ii) use of "envelopes" or" sealed envelopes
iii) simply stating that the study was randomised with no further details

**3) Inadequate**
i) by using case numbers, dates of birth, age group, alternation or date of referral and other similar methods
ii) use of any other system in which allocation can be known in advance such as pre-printed lists of random numbers or open computer allocation lists.

### Data synthesis

Data for each trial were entered into MS Excel spreadsheets and analysed using Revman version 4.2.8. Trials of supportive interventions often measure outcomes using ordinal rating scales. Where the rating scales used in the trials had more than seven categories the data were treated as continuous outcomes and normally distributed.

Many studies failed to report change from baseline or only reported post-intervention means, number of patients and standard deviations. Consequently, we assumed (given satisfactory randomisation) that control and experimental trial arms were comparable at baseline and compared post intervention means or event rates for those studies with sufficiently homegenous interventions.

For the meta-analysis of results of trials we used the weighted mean difference (WMD) when the pooled trials used the same rating scale or test, and the standardised mean difference (SMD) when they used different rating scales or tests. The duration of the trials varied considerably but did not necessitate dividing the meta analysis into smaller time periods with separate meta-analyses for each period. For binary outcomes, such as improvement or no improvement, the odds ratio (OR) was used to measure treatment effect.

In all cases the overall estimate from a fixed-effects model was presented except in cases where there was evidence of substantial heterogeneity between trials in which case a random-effects model was used [17]. I^2 ^statistic was employed to assess the impact of heterogeneity on the meta-analyses; a result of 50% or greater was regarded as substantial [18]. Regardless of statistical heterogeneity meta analysis was only undertaken when the interventions were sufficiently similar in focus and process to allow pooling.

## Results

A total of 141 relevant titles and abstracts were provided to authors following the database search by the Editorial Group of the Cochrane Dementia and Cognitive Improvement Group. Following screening, 44 papers (Appendix 1) were included in the final review [A1–A44] and 4 classified as 'pending assessment' (no published results or ongoing) [19-22].

Interventions were classified as technology-based (n = 4), individual (n = 27) or group-based (n = 13). However, within each of these classifications the range of interventions varied considerably and were sometimes not mutually exclusive, for example some trials comprised both group and individual aspects. Pragmatic decisions were made where necessary regarding classification for the purposes of describing studies. However, for the meta-analysis, group intervention was compared to control and individual intervention compared to control, and combined group plus individual interventions were classified according to the predominant component of the intervention [see additional file 1].

### Technology-based interventions

**Figure 1:**
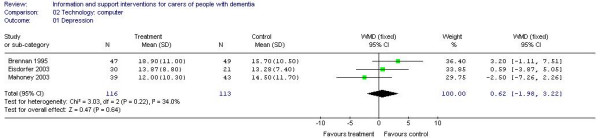
Technology-based computer interventions and depression.

Four studies were classified as technology based interventions. Goodman [A17] reported that the intervention led to significant improvement on outcomes of information, perceived social support and support satisfaction whereas Mahoney [A23] found no effect for the intervention on any of the assessed outcomes. Various subgroup analyses by trialists were undertaken, reporting significant benefits for the intervention [A1, A8, A23]. Effect estimate on outcome of depression for 3 trials (229 participants) [A1, A8, A23] using computer interventions was 0.62, however this did not reach statistical significance (95% CI -1.98 to 3.22) (Figure 1).

### Group-based interventions

Thirteen of the included studies were classified as group based interventions. Eight trials found significant benefits for the interventions compared to controls on some of the outcomes [A6, A9, A11, A19, A20, A24, A26, A38]. Five trials reported no differences between groups on any assessed outcomes [A13, A18, A25, A32, A43].

**Figure 2:**
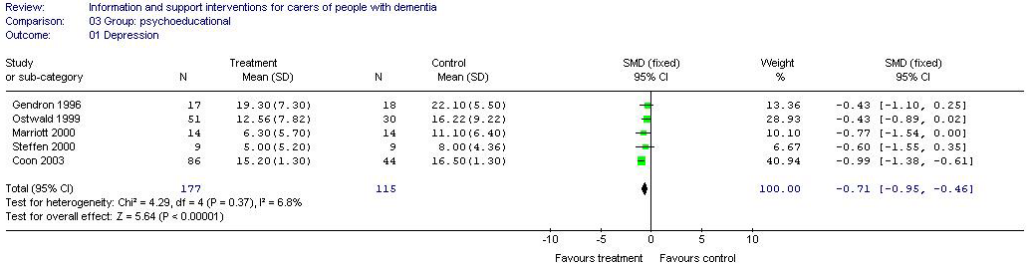
Group-based psychoeducational interventions and depression.

**Figure 3:**
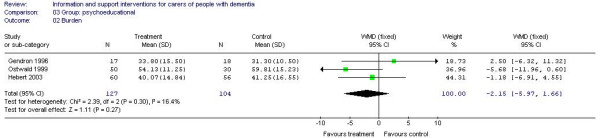
Group-based psychoeducational interventions and burden.

**Figure 4:**
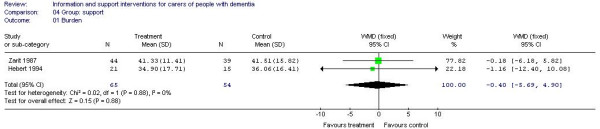
Group-based support interventions and burden.

Meta-analysis of 5 studies (292 participants) [A6, A13, A24, A32, A38] of psychoeducational interventions for caregiver depression estimated a statistically significant effect in favour of the intervention (-0.71, 95% CI -0.95 to -0.46) (Figure 2). Three studies (231 participants) [A13, A20, A32] of psychoeducational intervention assessed burden. There were no significant differences between the groups (effect estimate -2.15, 95% CI -5.97 to 1.66) (Figure 3). Meta-analysis of 2 studies of support interventions (119 participants) [A19, A43] revealed no significant differences in carer burden between the intervention and control groups (effect estimate -0.40, (95% CI -5.69 to 4.90) (Figure 4).

### Individual-based interventions

Twenty seven studies were classified as individual-based interventions. Ten trials found no difference between the groups in the main analysis of the assessed outcomes [A5, A12, A14, A29, A30, A33, A35, A36, A41, A44]. Twelve reported a significant effect for the intervention on some of the assessed outcomes [A3, A4, A7, A15, A16, A21, A22, A28, A31, A34, A39, A40] and one trial reported a significant effect in favour of the control group [A42]. Four studies reported results based on subgroup analyses [A2, A10, A27, A37].

**Figure 5:**
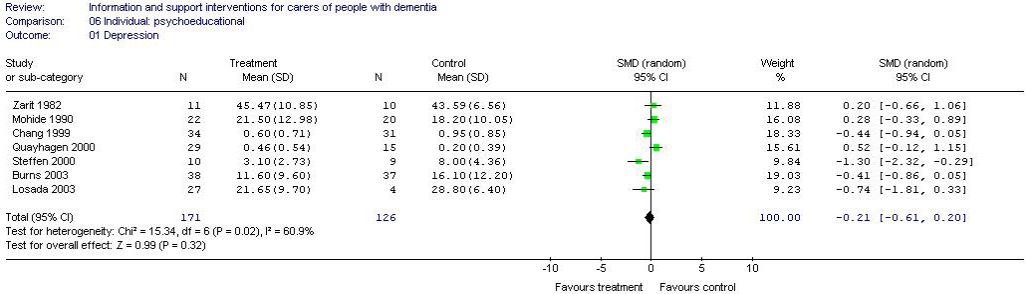
Individual-based psychoeducational interventions and depression.

**Figure 6:**
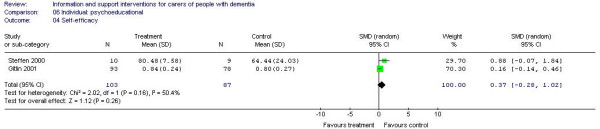
Individual-based psychoeducational interventions and self efficacy.

Meta-analysis of individual psychoeducational interventions was possible for outcomes of depression (7 trials; 297 participants) [A3, A4, A22, A29, A34, A38, A42] and self-efficacy (2 trials; 190 participants) [A14, A38]. For depression, the estimate of effect was -0.21 in favour of the intervention (95% CI -0.61 to 0.20) (Figure 5) and for self-efficacy 0.37 in favour of the control group (95% CI -0.28 to 1.02) (Figure 6) but neither estimate reached statistical significance.

### Methodological quality of included studies

The overall quality of included studies was poor. All the included studies were reported as being randomised, however for the majority of studies (n = 41) the method of randomisation was unclear (Category 2) and concealment of allocation was rare. The sample size calculations were poorly reported, with only two of the included trials citing an adequate a priori power calculation [A2, A24].

Over one third of the trials (n = 14) were stated as non-blinded which is perhaps not unusual given the nature of the interventions. However, over 40% did not provide any information on whether blinding (of the participants, the investigators or the outcome assessors) was employed. Only one trial [A37] was conducted in a double-blind manner where the investigators and subjects were blind to the group allocation. Twelve trials [A3, A6, A7, A18, A19, A20, A23, A24, A26, A34, A39, A40] stated that the outcome assessors were blind and one further trial noted that the participants were blind to group allocation [A12].

Most of the included studies reported attrition although this was not always done by group allocation, rather just an overall percentage of all the participants. Attrition ranged from none to 55%. Over two thirds of the studies did not use intention to treat analysis.

A number of the included studies selectively reported outcomes. This was primarily in the form of significant outcomes being reported but a lack of data for other outcomes was also apparent. In addition, many of the studies undertook post-hoc subgroup analyses, often where no significant main effect was detected. There was considerable variation in the length of follow-up amongst the trials although the majority had a maximum follow-up of up to 12 months.

## Discussion

### Main findings

Information and support interventions are delivered via a number of formats. Of these, only group interventions (underpinned by psychoeducational theoretical foundations) appear to positively impact on depression in caregivers. The extent to which these benefits are clinically (rather than just statistically) significant remains uncertain. Putting aside the difficulties of ascertaining clinical significance, considerable caution needs to be exercised in concluding that group based information and support can influence depression. The aetiology of depression is uncertain [23] but psychological, physical and social factors are often cited as risk factors. Given the lack of impact of information and support interventions on other outcomes that might be expected to correlate with depression (such as the sense of burden that is assumed to accompany caregiving) the apparent impact on depressive symptoms should be interpreted tentatively. Indeed, other researchers [24] have also found that depression is "surprisingly" unrelated to a sense of burden and satisfaction with life. As well as the difficulties in unpacking the relationship between depression and burden, the interface between statistical and clinical significance presents challenges for clinicians. Specifically, the standardised mean difference presents two major obstacles to interpreting the apparent effects. Firstly, the quantity of the *number of standard deviations between the means *– the SMD – is not directly useable for decision making [25]. Secondly, the reported trials all used different ordinal scales as the basis for measurement which increases this difficulty. Interpreting the clinical significance of a point estimate shift on a single scale is often difficult, doing so for an average effect size across four different scales (with their associated sensitivities and specificities) is even more precarious. Some studies (A10 and A24) sampled carers on the basis of their psychological morbidity and it might be expected such studies would illustrate the differential effects of interventions. However, whilst – of the five studies included in the meta analysis of group psychoeducational interventions – Marriot [A24] had the second largest effect size (-0.77) it was still only 0.06 SMD greater than the mean for the group (-0.71) and only just statistically significant (95% confidence interval -1.54 – 0.00). This was also a study with only 14 carers in each arm of the comparison. It was not possible to include the Gallagher-Thompson [A10] study in any meta analysis as it presented analysis on the basis of subgroups.

### Overall completeness and applicability of evidence

Providing information and support alone is rare in interventions for caregivers of people with dementia – or at least those that are subject to randomised clinical trial evaluation. Often the provision of information and what might be termed 'support' is packaged as part of broader intervention built on theoretical principles of problem solving, coping or psycho-education/training. There are also elements of information provision that are missing from the evidence base, the most obvious being the simple provision of information as a passive activity, for example mass media advertising of support groups, or the targeted provision of educational materials alone.

One of the strengths of pragmatic clinical trials is the commitment to replicating the 'real life' conditions of the interventions. Often the subjects recruited for clinical trials of interventions designed to support caregivers are from the lists of support organisations such as the Alzheimer's Society or from local healthcare clinics. Consequently, the vast majority of the trials in this review fail to capture the effect of the interventions on those caregivers who are not already in receipt of some form of supportive interventions or scrutiny. Similarly, reporting reasons for dropping out of the trials is the exception rather than the norm in the included studies. Therefore the possibility exists that interventions may not be universally popular with caregivers and that caregivers are leaving interventions for reasons that are counterintuitive and hidden.

A further difficulty with the evidence base is the heterogeneity in the outcomes (and associated measures) used in each trial. With the exception of burden and depression the numbers of outcomes measured in studies is vast. Where studies share a common outcome (such as depression) the measures used are often very different (including condition specific measures or measures of general health). Comparing the results of several trials of similar interventions would be made much easier if researchers in the area of supportive interventions would work with those outcomes most sensitive to change and adopt some common shared measures. Initiatives such as the US REACH programme [26] go someway to addressing this limitation. Perhaps the most significant weakness of the evidence base is the short period of follow up in most clinical trials. Despite the median survival time from diagnosis to death being over 8 years for many dementia patients [27] the majority of studies follow-up only for 12 months. Follow up periods are also important as it is quite possible that caregiver needs and responses to interventions will alter according to their stage in the caregiving trajectory [28].

### Quality of the evidence

Systematically reviewing trials in the area of support for caregivers of people with dementia is made difficult by the overwhelmingly poor quality of the available evidence and the lack of adherence to what might be termed 'best practice' in trial-based evaluation of effectiveness. The evidence base is characterised by studies in which well described randomisation, blinding of allocation or outcome assessment, intention-to-treat analysis and restriction of analysis to primary outcomes are the exceptions. These indicators of quality are important given the possible impact on the estimated effect size – up to 30% where randomisation alone is unclear [16].

Non blind outcome assessment increases effect size [16] and this is more likely for subjective outcomes such as burden ('objective' outcomes such as mortality are less likely to be affected) [29]. Unfortunately, most of the outcomes measured in the area of support for caregivers (such as burden or depression) rely on self report questionnaires – a characteristic which leaves them more prone to social desirability bias.

Very few studies made use of explicit a priori power calculations to ensure that sample sizes were large enough to avoid type 1 and type 2 errors and thus spurious claims of effect. Whilst statistical heterogeneity in the meta-analyses subgroups was rare there was considerable heterogeneity in intervention content. This was particularly so in the extent to which interventions were informed by theoretical frameworks. Some interventions (such as Buckwalter [A2] and Chang [A4]) were designed around explicit models of behaviour and interaction with the caregiving environment, whilst others such as Goodman [A17] appeared more pragmatic.

The poor average quality of the 44 studies included in the review actively reduces the confidence with which conclusions can be drawn relating to effectiveness.

### Potential limitations and strengths of the review

This review focused only on randomised trials, taking into account the quality of the evidence involved in our analysis. This is a strength, in that it makes any findings more cautious, and also a possible limitation in that the wealth of non-random, quasi-experimental, evidence available may yield clues regarding the underlying effects of interventions. However, systematic reviews [10,11] and meta analysis [30] which have included non-randomised studies suggest, as we do, that there is little evidence to support the idea of *universal *effectiveness of interventions to improve the lives of caregivers of people with dementia. New evidence in this area is emerging all the time and given the relatively small size of trials in each subgroup it is likely that a large, high quality, clinical trial in a single subgroup may significantly alter any future analysis.

Whilst the quantitative results yielded inconclusive findings, many of the studies we examined reported a number of qualitative findings that suggest that a broader approach to the elucidation of the effects of interventions designed to help caregivers might yield some meaningful results and areas for more rigorous evaluation in future research. For example, Robinson [A36] highlights the ability to share perceptions and solve care-related problems within caregiver groups as an important qualitative dimension of the experience of receiving the intervention. Brennan [A1] in her report of the effectiveness of a computer network found that one of the reasons for drop-out was that 'the computer' frightened a subject. Ongoing work on systematically reviewing qualitative findings should help elucidate these experiences and could help inform the development of measures of effect with greater internal validity.

## Conclusion

The recent *Dementia UK *report [3] recommends carer support packages and in particular psychological therapies that include carer training and support groups. Our review suggests that there is no evidence that information and support-based interventions for caregivers of people with dementia are uniformly effective. Very limited evidence exists supporting the argument that information and support, in the context of group psychoeducational approaches, might have a statistically significant positive effect on depression. However, the small size of the studies involved and the considerable variations in content within the subgroups mean that this conclusion should be interpreted very cautiously. Moreover, despite the statistical significance of the result, the clinical significance of the improvement remains uncertain. The relationship to any possible harms (such as an increased sense of burden as a result of having to receive the intervention) also remains unknown.

For any policy maker or service planner in the area of carer support there are fundamental questions that require answers: "which carers are most likely to benefit from our service?"; "in what circumstances will this package be effective?"; "at what stage in the caring trajectory should we introduce this service to deliver the most benefit to carers?". Our research suggests that evidence based decision makers will have to wait some time for the answers to these vital questions. The quality of the existing evidence base, as revealed in this review, simply does not allow evidence-based estimates of such effects. Caring is an emotive subject, and addressing the possibility that what is provided by health services to help carers may not be as beneficial as we would like to believe is a challenging research agenda; but perhaps more than ever, there really is a need for radical scrutiny of what exists and what we can learn from well constructed evaluations.

## Competing interests

The author(s) declare that they have no competing interests.

## Authors' contributions

**CAT**: study design, screening of papers and data extraction of included studies, data analysis, preparation of manuscript.

**KS**: study design, screening of papers and data extraction of included studies, preparation of manuscript.

**JH**: screening of papers and data extraction of included studies, data analysis, preparation of manuscript sections and commented on other sections.

**YFB**: data extraction of included studies and commented on manuscript.

**CB**: data extraction of included studies, contribution to analysis and commented on manuscript.

**JA**: moderator for disagreements relating to inclusion/exclusion of studies, data extraction and commented on manuscript.

## Appendix 1: References to included studies and linked papers (+)

A1. Brennan FP, Moore SM, Smyth KA: **The effects of a special computer network on caregivers of persons with Alzheimer's disease. ***Nursing Research *1995, 44(3): 166–72

+ Bass DM, McClendon MJ, Brennan PF, McCarthy C: **The buffering effect of a computer support network on caregiver strain. ***Journal of Aging & Health *1998, 10(1): 20–43

+ McClendon MJ, Bass DM, Brennan PF, McCarthy C: **A computer network for Alzheimer's caregivers and use of support group services**. *Journal of Mental Health and Aging *1998, 4(4): 403–20

A2. Buckwalter KC, Gerdner L, Kohout F, Richards Hall G, Kelly A, Richards B, Sime M: **A nursing intervention to decrease depression in family caregivers of persons with dementia**. *Archives of Psychiatric Nursing *1999, 13(2): 80–8

+Hall GR: **Testing the PLST model with community-based caregivers. *Dissertation ****Abstracts International: Section B: the Sciences and Engineerin *g 1999, 60: 577–B

+Stolley JM, Reed D, Buckwalter KC: **Caregiving appraisal and interventions based on the progressively lowered stress threshold model**. *American Journal of Alzheimer's Disease and Other Dementias *2002, 17(2): 110–20

A3. Burns R, Nichols LO, Martindale Adams J, Graney MJ, Lummus A: **Primary care interventions for dementia caregivers: 2-year outcomes from the reach study**. *Gerontologist *2003, 43 (4): 547–55

A4. Chang BL: **Cognitive-behavioral intervention for homebound caregivers of persons with dementia**. *Nursing Research *1999, 48(3): 173–81

A5. Chu P, Edwards J, Levin R, Thomson J: **The use of clinical case management for early stage Alzheimer's patients and their families**. *American Journal of Alzheimer's Disease *2000, 15(5): 284–90

A6. Coon DW, Thompson L, Steffen A, Sorocco K, Gallagher Thompson D: **Anger and depression management: psychoeducational skill training interventions for women caregivers of a relative with dementia. ***Gerontologist *2003, 43(5): 678–89

A7. Corbeil RR, Quayhagen MP, Quayhagen M: **Intervention effects on dementia caregiving interaction. A stress-adaptaion modeling approach**. *Journal of Ageing and Health *1999, 11(1): 79–95

A8. Eisdorfer C, Czaja SJ, Loewenstein DA, Rubert MP, Arguelles S, Mitrani VB, Szapocznik J: **The effect of a family therapy and technology-based intervention on caregiver depression. ***Gerontologist *2003, 43(4): 521–31

A9. Fung W, Chien W: **The effectiveness of a mutual support group for family caregivers of a relative with dementia**. *Archives of Psychiatric Nursing *2002, 16(3): 134–44

A10. Gallagher-Thompson D, Steffen AM: **Comparative effects of cognitive-behavioral and brief psychodynamic psychotherapies for depressed family caregivers. ***Journal of Consulting and Clinical Psychology *1994, 62(3): 543–9

A11. Gallagher-Thompson D, Lovett S, Rose J, McKibbin C, Coon D, Futterman A, Thompson LW: **Impact of psychoeducational interventions on distressed family caregivers**. *Journal of Clinical Geropsychology *2000, 6(2): 91–110

A12. Garand L, Buckwalter KC, Lubaroff D, Tripp-Reimer T, Frantz RA, Ansley TN: **A pilot study of immune and mood outcomes of a community-based intervention for dementia caregivers: the PLST intervention. ***Archives of Psychiatric Nursing *2002, 16(4): 156–67

A13. Gendron C, Poitras L, Dastoor DP, Perodeau G: **Cognitive-behavioral group intervention for spousal caregivers: findings and clinical considerations**. *Clinical Gerontologist *1996, 17(1): 3–19

A14. Gitlin LN, Corcoran M, Winter L, Boyce A, Hauck WW:**A randomized, controlled trial of a home environmental intervention: effect on efficacy and upset in caregivers and on daily function of persons with dementia**. *Gerontologist *2001, 41(1): 4–14

A15. Gitlin LN, Belle SH, Burgio LD, Czaja SJ, Mahoney D, Gallagher Thompson D, Burns R, Hauck WW, Zhang S, Schulz R, Ory MG, REACH Investigators: **Effect of multicomponent interventions on caregiver burden and depression: the reach multisite initiative at 6-month follow-up. ***Psychol Aging *2003, 18(3): 361–74

A16. Gitlin LN, Hauck WW, Dennis MP, Winter L: **Maintenance of effects of the home environmental skill-building program for family caregivers and individuals with Alzheimer's disease and related disorders. ***Journals of gerontology: Series A, Biological sciences and medical sciences *2005, 60(3): 368–74

A17. Goodman CC, Pynoos J: **A model telephone information and support program for caregivers of Alzheimer's patients**. *Gerontologist *1990, 30(3): 399–404

+Goodman C: **Evaluation of a model self-help telephone program: impact on natural networks. ***Social Work *1990, 35(6): 556–62

A18. Haley WE, Brown SL, Levine EG: **Experimental evaluation of the effectiveness of group intervention for dementia caregivers**. *Gerontologist *1987, 27: 376–82

A19. Hebert R, Leclerc G, Bravo G, Girouard D, Lefrancois R. **Efficacy of a support group programme for care-givers of demented patients in the community: A randomized controlled trial**. *Archives of Gerontology and Geriatrics *1994, 18(1): 1–14

+Hebert R, Girouard D, Leclerc G, Bravo G, Lefrancois R: **The Impact of a support group programme for care-givers on the institutionalisation of demented patients**. *Archives of Gerontology and Geriatrics *1995, 20: 129–34

A20. Hebert R, Levesque L, Vezina J, Lavoie JP, Ducharme F, Gendron C, Préville M, Voyer L, Dubois MF: **Efficacy of a psychoeducative group program for caregivers of demented persons living at home: a randomized controlled trial**. J *Gerontol B Psychol Sci Soc Sci*. 2003, 58B(1): S58–67

A21. Logiudice D, Waltrowicz W, Brown K, Burrows C, Ames D, Flicker L: **Do memory clinics improve the quality of life of carers? **A randomized pilot trial. *International Journal of Geriatric Psychiatry *1999, 14: 626–32

A22. Losada Baltar A, Izal Fernandez de Troconiz M, Montorio Cerrato I, Marquez Gonzalez M, Perez Rojo G: **Differential efficacy of two psychoeducational interventions for dementia family caregivers**. *Rev Neurol *2004, 38(8): 701–8

A23. Mahoney DF, Tarlow BJ, Jones RN.**Effects of an automated telephone support system on caregiver burden and anxiety: findings from the REACH for TLC intervention study. ***Gerontologist *2003, 43(4): 556–67

+Mahoney DF, Tarlow B, Sandaire J: **A computer-mediated intervention for Alzheimer's caregivers. **Computers in Nursing 1998, 16(4): 208–16

A24. Marriott A, Donaldson C, Tarrier N, Burns A: **Effectiveness of cognitive-behavioural family intervention in reducing the burden of care in carers of patients with Alzheimer's disease. ***British Journal of Psychiatry *2000, 176: 557–62

A25. Martin Cook K, Remakel Davis B, Svetlik D, Hynan LS, Weiner MS: **Caregiver attribution and resentment in dementia care. ***American Journal of Alzheimer's disease and other dementias *2003, 18(6): 366–74

A26. McCurry SM, Logsdon RG, Vitiello MV, Teri L: **Successful behavioral treatment for reported sleep problems in elderly caregivers of dementia patients: A controlled study. ***Journals of Gerontology Series B: Psychological Sciences and Social Sciences *1998, 53B(2): 122–9

A27. Mittelman MS, Ferris SH, Shulman E, Steinberg G, Ambinder A, Mackell JA, Cohen J: **A comprehensive support program effect on depression in spouse-caregivers of AD patients**. *Gerontologist *1995, 35(6): 792–802

+Mittelman MS, Ferris SH, Shulman E, Steinberg G, Levin B: **A Family Intervention to Delay Nursing Home Placement of Patient With Alzheimer Disease**. *JAMA *1996, 276(21): 1725–31

+Mittelman MS, Ferris SH, Steinberg G, Shulman E, Mackell JA, Ambinder A, Cohen J: **An Intervention That delays Institutionalization of Alzheimer's Disease Patients: Treatment of Spouse-Caregivers. ***Gerontologist *1993, 33(6): 730–40

A28. Mittelman MS, Roth DL, Coon DW, Hayley WE: **Sustained benefit of supportive intervention for depressive symptoms in caregivers of patients with Alzheimer's disease. ***American journal of psychiatry *2004, 161(5): 850–6

A29. Mohide EA, Pringle DM, Streiner DL, Gilbert JR, Muir G, Tew M.**A randomized trial of family caregiver support in the home management of dementia**. *Journal of American Geriatrics Society *1990, 38: 446–54

+Drummond MF, Mohide EA, Tew M, Streiner DL, Pringle DM, Gilbert JR: **Economic evaluation of a support program for caregivers of demented elderly. ***International Journal of Technology Assessment in Health Care *1991, 7: 209–19

A30. Newcomer R, Yordi C, DuNah R, Fox P, Wilkinson A: **Effects of the Medicare Alzheimer's disease demonstration on the use of community-based services**. *Health Services Research *1999, 31(3): 645–67

A31. Nobili A, Riva E, Tettamanti M, Lucca U, Liscio M, Petrucci B, Porro GS: **The effect of a structured intervention on caregivers of patients with dementia and problem behaviors: a randomized controlled pilot study. ***Alzheimer Dis Assoc Disord *2004, 18(2): 75–82

A32. Ostwald SK, Hepburn KW, Caron W, Burns T, Mantell R: **Reducing caregiver burden: a randomized psychoeducational intervention for caregivers of persons with dementia**. *Gerontologist *1999, 39(3): 299–309

+Hepburn KW, Tornatore J, Center B, Ostwald SW: **Dementia family caregiver training: affecting beliefs about caregiving and caregiver outcomes**. *Journal of the American Geriatrics Society *2001, 49(4): 450–7

A33. Pillemer K, Suitor JJ: **Peer support for Alzheimer's caregivers: is it enough to make a difference? ***Research on Aging *2002, 24(2): 171–92

A34. Quayhagen MP, Quayhagen M, Corbeil RR, Hendrix RC, Jackson JE, Snyder L, Bower D: **Coping with dementia: Evaluation of four non pharmacologic interventions. ***Int Psychogeriatr *2000, 12(2): 249–65

A35. Roberts J, Browne G, Milne C, Spooner L, Gafni A, Drummond-Young M, LeGris J, Watt S, LeClair K, Beaumont L, Roberts J. **Problem-solving counselling for caregivers of the cognitively impaired: effective for whom? ***Nursing Research *1999, 38(3): 162–71

A36. Robinson K, Yates K:**Effects of two caregiver-training programs on burden and attitude toward help. ***Archives of Psychiatric Nursing *1994, 8(5): 312–9

A37. Schmidt GL, Bonjean MJ, Widem AC, Schefft BK, Steele DJ: **Brief psychotherapy for caregivers of demented relatives: comparison of two therapeutic strategies**. *Clinical Gerontologist *1988, 7(3/4): 109–25

A38. Steffen AM: **Anger management for dementia caregivers: A preliminary study using video and telephone interventions**. *Behavior Therapy *2000, 31(2): 281–99

+Steffen AM: **Anger management for dementia caregivers: innovative solutions using video and telephone interventions**. In: *The Gerontological Society of America 52nd Annual Scientific Meeting*, 19–23 November 1999, 39 (Special Issue 1)

A39. Sutcliffe C, Larner S: **Counselling carers of the elderly at home: a preliminary study. ***British Journal of Clinical Psychology *1988, 27(2): 177–8

A40. Teri L, Logsdon RG, Uomoto J, McCurry SM: **Behavioral treatment of depression in dementia patients: a controlled clinical trial**. *Journal of Gerontology *1997, 52B(4): 159–66

A41. Weinberger M, Gold DT, Divine GW, Cowper PA, Hodgson LG, Schreiner PJ, George LK: **Social service interventions for caregivers of patients with dementia: impact on health care utilization and expenditures. ***Journal of American Geriatrics Society *1993, 41: 153–6

A42. Zarit SH, Zarit JM, Reever LE. **Memory training for severe memory loss: effects on senile dementia patients and their families**. *Gerontologist *1982, 22(4): 373–7

A43. Zarit SH, Anthony CR, Boutselis M: **Interventions with caregivers of dementia patients: Comparison of two approaches**. *Psychology and Aging *1987, 2(3): 225–32

+Whitlach CJ, Zarit SH, Von Eye A: **Efficacy of interventions with caregivers: a reanalysis. ***Gerontologist *1991, 31(1): 9–14

A44. Zimmer JG, Eggert GM, Chiverton P: **Individual versus team case management in optimizing community care for chronically ill patients with dementia**. *Journal of Ageing and Health *1990, 2(3): 357–72

## Pre-publication history

The pre-publication history for this paper can be accessed here:



## Supplementary Material

Additional file 1 details of the included studies. The spreadsheet includes details of the interventions and controls, their duration, the method of the included study, the means of allocation, outcomes reported and reported results. Click here for file
